# Management of Traumatology Patients During the Coronavirus (COVID-19) Pandemic: Experience in a Hub Trauma Hospital in Northern Italy

**DOI:** 10.1007/s43465-020-00282-5

**Published:** 2020-10-23

**Authors:** Fabio D’Angelo, Luca Monestier, Giovanni De Falco, Michael Mazzacane, Placido Stissi

**Affiliations:** 1 Division of Orthopaedics and Traumatology, Department of Biotechnologies and Life Sciences (DBSV), ASST Dei Sette Laghi, University of Insubria, Varese, Italy; 2 Division of Orthopaedics and Traumatology, ASST Sette Laghi, Varese, Italy; 3 Residency Program in Orthopedics and Trauma, Division of Orthopaedics and Traumatology, Department of Biotechnologies and Life Sciences (DBSV), ASST dei Sette Laghi, University of Insubria, Varese, Italy

**Keywords:** COVID-19, Trauma, Proximal femoral fracture, Management, Pandemic

## Abstract

**Background:**

As a result of the uncontrolled spread of the COVID-19 virus infection, a health reorganization according to the “hub and spoke” model was necessary. The purpose of the article was to document the adopted corporate protocol and describe the management of the traumatized patient in a Hub center.

**Methods:**

Our hospital has been identified as one of the three regional Hubs for polytrauma and major traumas, requiring suitable pathways to receive confirmed or suspected COVID-19-positive patients, from the emergency room entrance to the operating room, and finally to the inpatient ward or ICU. From February 23th to April 30th 2020 we analyzed the total number of trauma patients hospitalized and the number of femoral neck fractures surgically treated within 48 h; the data were then compared with the corresponding period of the previous year.

**Results:**

There has been a reduction in the overall number of traumas as a result of government restraint measures. Total occupancy time in the operating theater has increased, but not drastically considering dressing procedures and anesthesia (carried out inside the operating room). The number of patients with femoral neck fractures surgically treated within 48 h (none of the COVID-19-positive patients) decreased from 83.33 to 58.70%, but only slightly lower than the Italian pre-COVID average of 64.70%.

**Conclusions:**

The correct management of the hospital and the meticulous organization of the traumatized patient have made it possible to contain the potential negative effects on the medical care quality during this unexpected and severe health emergency.

## Background

COVID-19 is the infectious disease caused by the SARS-CoV-2 coronavirus. As the infection has spread to most of the world, the World Health Organization declared this novel outbreak a pandemic on March 11th, 2020.

On April 30th, Italy has been one of the most affected countries in the whole world with 205,463 total cases and 27,967 deaths. Most cases occurred in Lombardy, a northern district with about 10 million inhabitants: 71,256 cases (34.7% of the national amount) and 13,106 deaths (46.9% of the national amount), with a 1.7% admittance rate to intensive care unit (ICU) and 19.7% hospitalization rate [1]. Therefore, a change in regional healthcare organization and a rationalization of health resources were necessary to limit the virus outbreak: thus, the elective surgery has been suspended from February 23th 2020, in order to allow to free up beds for COVID-19 patients.

On March 8th 2020, the regional government decided to redirect patients affected by COVID-19 related severe acute pathologies to selected centers, creating a “Hub-and-Spoke” organization.

Regional hubs were selected for major trauma: orthopedic, neurosurgical, cardiologic, neurologic or vascular non-deferrable emergencies. These hospitals must guarantee a 24 h/7 days service, Intensive Care Unit beds and different pathways between COVID-19-positive and -negative patients. Minor surrounding hospitals (spokes) cooperate with these hubs for a structured and integrated patient management.

Our hospital (ASST Sette Laghi, Varese, Lombardy, Italy) was selected as one of the three major Hubs in Lombardy district for polytrauma and major traumatic emergencies.

We aim to share our experience about the management of cases during the past 2-month period of COVID-19 pandemic, describing our Hub organization and pathways for traumatic patients.

## Methods

### Population

A prospective cohort study with a retrospective control group was undertaken. Data on each patient admitted to our hospital with an admission diagnosis of a traumatic pathology requiring surgical treatment between 23rd February and 30th April 2020 were collected prospectively. These included demographics, diagnosis, type of surgery, and time interval between admission and surgery. This cohort was compared to a retrospective cohort of patients admitted for the same reasons in the same period the previous year (23rd February and 30th April 2019).

### Algorithm Adopted for Trauma Patients

Traumatology patients requiring surgical treatment may reach our Hub in two ways.

First, a direct access into the hub from the territory: in the emergency room the patient undergoes clinical evaluation, primary imaging procedures, hemodynamic stabilization, and orthopedic examination, and a COVID-19 swab is immediately performed (result is normally available within 6–8 h).

Second, the patient could be evaluated in a spoke hospital and then centralized to the hub, in case of a trauma emergency, or a COVID-19 positive case, or if the patient’s general health conditions require a recovery in the ICU during the peri-operative time.

**Fig. 1:**
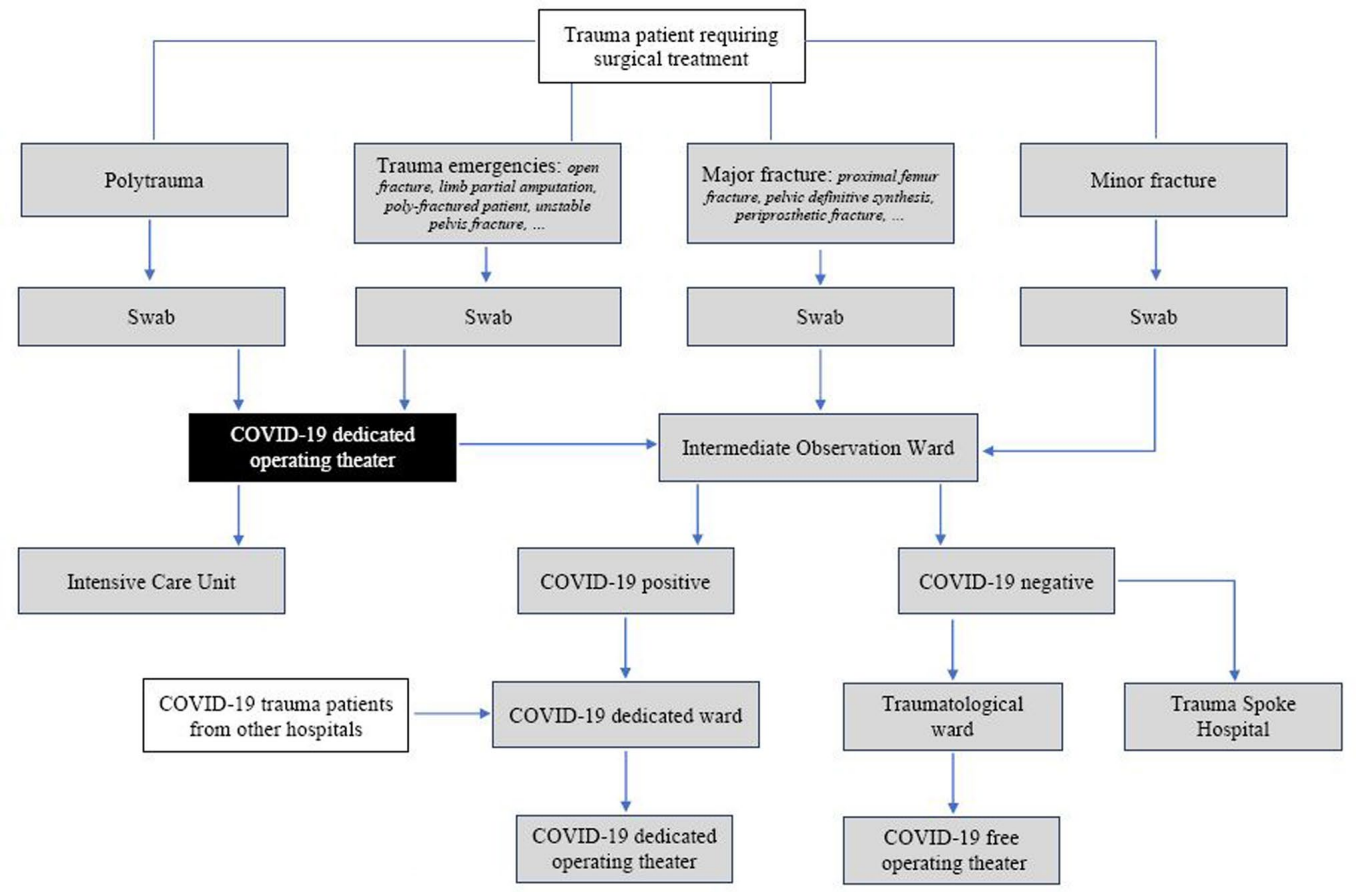
Flow chart about organization at our hub

We distinguish five different typologies of trauma patients; for each case, we follow an algorithm as explained in Fig. 1:

- *Polytrauma* refers to a trauma patient whose injuries involve multiple body regions (organs or apparatus) with existing or potential life-threatening condition. After clinical evaluation in the emergency room, primary radiological diagnostic procedures and hemodynamic stabilization, a COVID-19 swab is performed directly in the emergency room; a fast and priority access to a dedicated COVID-19 operating theater, with a 24 h/7 days available team, is carried out (see Fig. 1); after surgery, the patient goes strictly to Intensive Care Unit (ICU) or to an Intermediate Observation Ward (IOW) for postoperative care;
- *trauma emergencies* (open fracture, partial or complete limb amputation, poly-fractured patient, unstable pelvis fracture) COVID-19 swab is executed in the emergency room and the patient undergoes surgical treatment in a dedicated operating theater (see Fig. 1); afterward, the patient goes to ICU or IOW for postoperative care;
- *patients with major fractures* (proximal femur fracture, diaphyseal fracture, pelvis synthesis, periprosthetic fracture, fractures with dislocation) *that need a time-dependent surgical treatment* COVID-19 swab is executed in the emergency room and the patient goes to IOW pending the results. If COVID-19 positive, the patient undergoes surgical treatment in a dedicated operating theater (see Fig. 1); afterward, the patient is admitted to a specific COVID-19 ward for postoperative care; if negative, the patient goes to a COVID-19 free trauma surgical theater and then admitted to the Trauma Unit for the postoperative period;
- *patients with minor fractures (*shoulder, forearm, wrist, ankle, foot or forearm fractures, etc.*) that need a scheduled surgical treatment* COVID-19 swab is executed in the emergency room and the patient goes to IOW pending the results. If COVID-19 positive, the patient undergoes surgical treatment in a dedicated operating theater (see above); afterward, the patient is transferred to a specific COVID-19 ward for postoperative care; if negative, the patient is carried to a minor COVID-19 free trauma hospital (spoke) to undergo surgery, and
- *major/minor trauma in confirmed COVID-19-positive patients from other hospitals* the patient is transferred to a specific COVID-19 ward and undergoes surgical treatment in a dedicated operating theater (see Fig. 1).

We strongly highlighted the importance to create the Intermediate Observation Unit: this permits to create a filter and different pathways for COVID-19-positive or -negative patients, and not to perform surgical treatments in the same operative rooms.

Moreover, the possibility to hospitalize COVID-19-positive and -negative patients in different wards, using dedicated elevators and corridors, is fundamental to reduce the risk of nosocomial COVID-19 infections in already traumatized patients.

### Dedicated COVID-19 Operating Theater

At our hospital, we strictly follow the recommendations, indicated by the regional health government indicated on March 18th, and those shared by the World Health Organization on April 6th. Given the potential spread of COVID-19, all the health workers (doctors, nurses, support workers) should pay meticulous attention in the operative room.

Dedicated pathways (e.g. corridors, elevators) have been created in our hub for COVID-19-positive patients requiring surgical treatment for trauma.

In a filter anteroom, medical personnel perform accurate hand hygiene and successively wear personal protective equipment (PPE), in the following order:

- Sterile second-skin-gloves, which are maintained until the end of undressing procedure;
- neck and head protective equipment;
- face shield or protective glasses;
- protection for shoes;
- first sterile gown, for surgical procedures;
- mono-use FFP2 / N-95 mask;
- second sterile skin gloves, for surgical procedures, and
- second sterile gown and third skin gloves, which are used only for positioning the patient and are removed before surgery.

Using these protocols, we believe the most protection is given to limit the risk of SARS-CoV-2 transmission between patients and health workers.

Anesthetic procedures are performed directly in the operating theater, and close attention must be paid due to droplets or aerosol production. During these procedures, only anesthesiologists and dedicated nurses are present, while the rest of the personnel remain just outside the room.

Once surgery has finished, the undressing procedure is performed in the anteroom, under supervision of dedicated professionals: given the high risk to self-contamination or exposure of other health workers, PPE had to be removed accurately and in the correct sequence following an established protocol.

Last, a C-arm fluoroscopy for intra-operative x-rays is dedicated for this surgical room: it is not allowed to use this machine outside COVID-19 dedicated surgical theaters or for COVID-19-negative patients.

## Results

During the past 2 months, 134 patients (62 males and 72 females with a mean age of 64.23 years, range 4–99) were admitted at our hospital with a diagnosis of fracture. Of these, 28 (20.90%) were transferred to our spoke hospitals. According to our protocol, all patients were tested for COVID-19: 4 (2.99%) resulted COVID-19-positive, while others were negative. According to our protocol, nine patients (6.72%) with trauma emergencies were treated in COVID dedicated surgical room even if their swabs resulted negative after surgery. The diagnosis of these cases was as follows: three pediatric fractures, two polyfractured patients, one medial proximal femoral fracture in a 46-year-old patient, one open Gustilo type-2 tibial fracture, one closed high-energy fracture of the tibia, and an “open-book” injury of the pelvis.

During the same period in the previous year, 177 patients (69 males and 108 females with a mean age of 67.35 years, range 7–102) were admitted at our hospital with a diagnosis of fracture. Proximal femoral fractures represent, respectively, 34.32% (46/134) in 2020 and 30.50% (54/177) in 2019.

The comparison between the surgical activity at our Institution in a 2-month period during COVID-19 disease and the same months during 2019 is shown in Table 1.

**Table 1:** Comparison of orthopedic and trauma activities between 2019 and 2020

	23rd February–30th April 2019	23rd February–30th April 2020
Elective surgery	212	0
Traumas (femoral neck fractures excluded)	123	88
Femoral neck fractures (< 48 h)	45	27
Femoral neck fractures (> 48 h)	9	15
Femoral neck fractures in COVID-19-positive patients (all treated after 48 h)	0	4
Total	389	134

One patient with several comorbidities died within the first post-operative week during COVID-19 outbreak: this case was treated for femoral neck fracture (> 48 h by trauma) and was COVID-19 free. In the same period of 2019 when only one case of death was reported within first post-operative week, patient was treated for femoral neck fracture (> 48 h by trauma) and was affected by pre-operative severe cardiological comorbidity. The data are shown in Table 2.

**Table 2:** Comparison of mortality between 2019 and 2020

	23rd February–30th April 2019	23rd February–30th April 2020
Traumas (femoral neck fractures excluded)	0	0
Femoral neck fractures (< 48 h)	0	0
Femoral neck fractures (> 48 h)	1	1
Femoral neck fractures in COVID-19-positive patients (all treated after 48 h)	–	0
Total	1	1

Concerning the implant of a hip arthroplasty for a femoral neck fracture, we report an average of 102 mins time-of-surgery in COVID-19-negative patients, significantly lower than the 149mins in COVID-19-positive patients.

## Discussion: Our Experience During COVID-19 Outbreak

COVID-19 disease represents an unprecedented challenge for worldwide national and regional health systems. Despite that most health efforts are correctly intended to limit the pandemic propagation, different surgical treatments could not be postponed: trauma, oncological, neurosurgical, vascular diseases must be treated rapidly [2–5].

As a result, also the treatment of trauma patients is affected during this period: non-urgent surgical procedures are delayed until swab results, the operating room time is prolonged due to protection protocols, and the total number of traumatology beds in the hospital are reduced, likewise to those in Intensive Care Unit dedicated to COVID-free patients.

As shown in Table 1, the decrease of the surgical activity due to pandemic has been about 65.55%. Trauma surgery has been also reduced significantly: 177 cases in 2019 versus 134 in 2020 (− 24.29%).

Italian Health Minister created the PNE (Piano Nazionale Esiti), an audit system to evaluate national hospitals based on clinical parameters and organizational aspects [6]. One of the parameters concerning the evaluation is the surgical treatment of femoral neck fractures within 48 h by the hospital access, in order to reduce mortality. The last report published in 2018 showed that 270 cases of femoral neck fracture were admitted to our institution, 3rd regional hospital by the number of cases, and that 85.68% of them were surgically treated within 48 h, while the mean of all Italian hospitals is 64.70%, classifying our Unit at the top-50 in Italy, 8th in Lombardy.

During the past 2 months of COVID-19 outbreak, we surgically treated 46 patients with femoral neck fractures (4 confirmed COVID-19-positive cases): 27 patients (58.70%) underwent surgery within 48 h. None of the COVID-19-positive patients were treated before 48 h from the fracture diagnosis.

We operated 54 patients during the same months in 2019, 45 of them (83.33%) before 48 h. Even if the total amount of cases with proximal femur fracture has not substantially reduced, the delayed treatment (> 48 h) percentage increased: 9 cases in 2019 (16.67%) versus 19 cases in 2020 (41.30%). This fact is related to the limited availability of dedicated orthopedic operating rooms.

Despite COVID-19 outbreak, the before-48 h-treatment of these patients at our Institution is a bit lower than the national average reported in Italian hospitals before the pandemic. Nonetheless, the delay in treating proximal femur fractures in elderly patients is shown to have detrimental effects and the risk of mortality arises significantly [7–9]. Among our patients, one patient (5.26%) treated after 48 h died within the first post-operative week: this case had several comorbidities and was COVID-19 free. In our population, the mortality in COVID-19-positive patients with a proximal femur fracture has been 0.00%, lower than 9.60% reported in a recent article [10]. Our result is deeply vitiated by the reduced sample of cases.

Last, the more extending dressing protocols and preoperative procedures in the surgical room, the longer time of surgery in COVID-19-positive patients than negative ones: we define the time-of-surgery from the moment the patient enters the surgical theatre to the moment he/she leaves the room (thus, anesthesiologic procedures, patient positioning, and surgical procedures are included).

The longer time of surgery in COVID-19-positive patients (102 mins vs 149 mins) for hip arthroplasty implant is presumably due to additional procedures necessary for limiting virus outbreak in operative room.

The main limitation of this study is that it is an observational study. It provides a picture of a limited timeframe and in a single hub reality.

## Conclusions

COVID-19 disease represents an unprecedented challenge also for Trauma Units in the world.

At our institutions, the results of the new organization were the following:

- A longer time-of-surgery in both confirmed cases and suspected ones but judged urgent by the trauma surgeon as open fractures or high-energy trauma;
- a delayed surgical treatment due to pending swab results in scheduled cases but time-dependent as proximal femoral fractures.

The correct management of the hospital and the meticulous organization of the traumatized patient have made it possible to contain the potential negative effects on the medical care quality during this unexpected and severe health emergency.

## Data Availability

All data generated or analyzed during this study are included in this published article.

## Abbreviations

COVID-19: COronaVIrus Disease-19
ICU: Intensive Care Unit

## References

[1] Italian Ministry of Health website—https://opendatadpc.maps.arcgis.com/apps/opsdashboard/index.html#/b0c68bce2cce478eaac82fe38d4138b1. Accessed 1 May 2020.

[2] Chang-Liang Z, Wang W, Murphy D, Po-Hui JH (2020). Novel coronavirus and orthopaedic surgery: early experiences from Singapore. The Journal of Bone and Joint Surgery. American Volume.

[3] Giorgi PD, Villa F, Gallazzi E, Debernardi A, Schirò GR, Crisà FM, Talamonti G, D’Aliberti G (2020). The management of emergency spinal surgery during the COVID-19 pandemic in Italy. The Bone and Joint Journal..

[4] Zoia C, Bongetta D, Veiceschi P, Cenzato M, Di Meco F, Locatelli D, Boeris D, Fontanella MM (2020). Neurosurgery during the COVID-19 pandemic: update from Lombardy, northern Italy. Acta Neurochirurgica (Wien).

[5] Randelli PS, Compagnoni R (2020). Management of orthopaedic and traumatology patients during the Coronavirus disease (COVID-19) pandemic in northern Italy. Knee Surgery Sports Traumatology Arthroscopy.

[6] Programma Nazionale Esiti website—www.pne.agenas.it. Accessed 1 May 2020.

[7] Ram GG, Govardhan P (2019). In-hospital mortality following proximal femur fractures in elderly population. The Surgery Journal (NY)..

[8] Mattisson L, Bojan A, Enocson A (2018). Epidemiology, treatment and mortality of trochanteric and subtrochanteric hip fractures: data from the Swedish Fracture Register. BMC Musculoskeletal Disorders.

[9] Chlebeck JD, Birch CE, Blankstein M, Kristiansen T, Bartlett CS, Schottel PC (2019). Nonoperative geriatric hip fracture treatment is associated with increased mortality: a matched cohort study. Journal of Orthopaedic Trauma.

[10] Muñoz Vives JM, Jornet-Gibert M, Camara-Cabrera J, Esteban PL, Brunet L, Delgado-Flores L, Camacho-Carrasco P, Torner P, Marcano-Fernàndez F (2020). Mortality rates of patients with proximal femoral fracture in a worldwide pandemic. JBJS.

